# The individualized treatment for minimally invasive repair of pectus carinatum in adolescent: a single center’ s retrospective study

**DOI:** 10.1186/s13019-024-02910-9

**Published:** 2024-08-16

**Authors:** Xudong Ran, Weijia Shen, Xin Li, Jianyi Liao, Hongliang Yuan, Hao Wang, Songhua Wu, Shuhan Rong

**Affiliations:** Cardiothoracic surgery department, children’ s hospital of Soochow university, No. 92 Zhongnan Street, Suzhou, 215000 Jiangsu Province China

**Keywords:** Pectus Carinatum, Minimally invasive repair, Adolescent, Abramson procedure, Nuss procedure, Individually

## Abstract

**Background:**

Pectus carinatum (PC) mainly present at the growth spurt time of the early teenage years or the puberty. Poor outer appearance is a major reason for seeking help for surgeons to increase self-confidence and self-esteem. At present, minimally invasive repair (MIR) is one of effective ways to correct the chest wall deformity. Therefore, there is great practical significance to conduct clinical research on MIR about the adolescent PC.

**Methods:**

We applied Abramson procedure in PC group or we applied Abramson procedure and Nuss procedure in PC/PE group. We retrospectively reviewed the results of 41 cases who underwent the surgical correction at our department from January 2020 to April 2023.

**Results:**

All the procedures were successfully done without severe complications. The median operation Time was 80 min in PC group while was 130 min in PC/PE group. The median LOS were 4 days in PC group while 5 days in PC/PE group. The median compression depth was 32 mm in PC group while 12 mm in PC/PE group. Postoperatively, there are some complications. All Pneumothorax patients being treated conservatively were found in 9 patients in two groups. One patient suffered overcorrection after operation. There were 3 patients suffered steel wires breakage in two groups. One patient reoperation postoperatively for the dislocation of the bar secondary to steel wires breakage.

**Conclusions:**

The Abramson procedure or Abramson procedure and Nuss procedure have good short-term results in repair PC and PC/PE. Select one or two procedures should be done individually based on whether the lower plane over depressed after Abramson procedure.

## Introduction

**Fig. 1:**
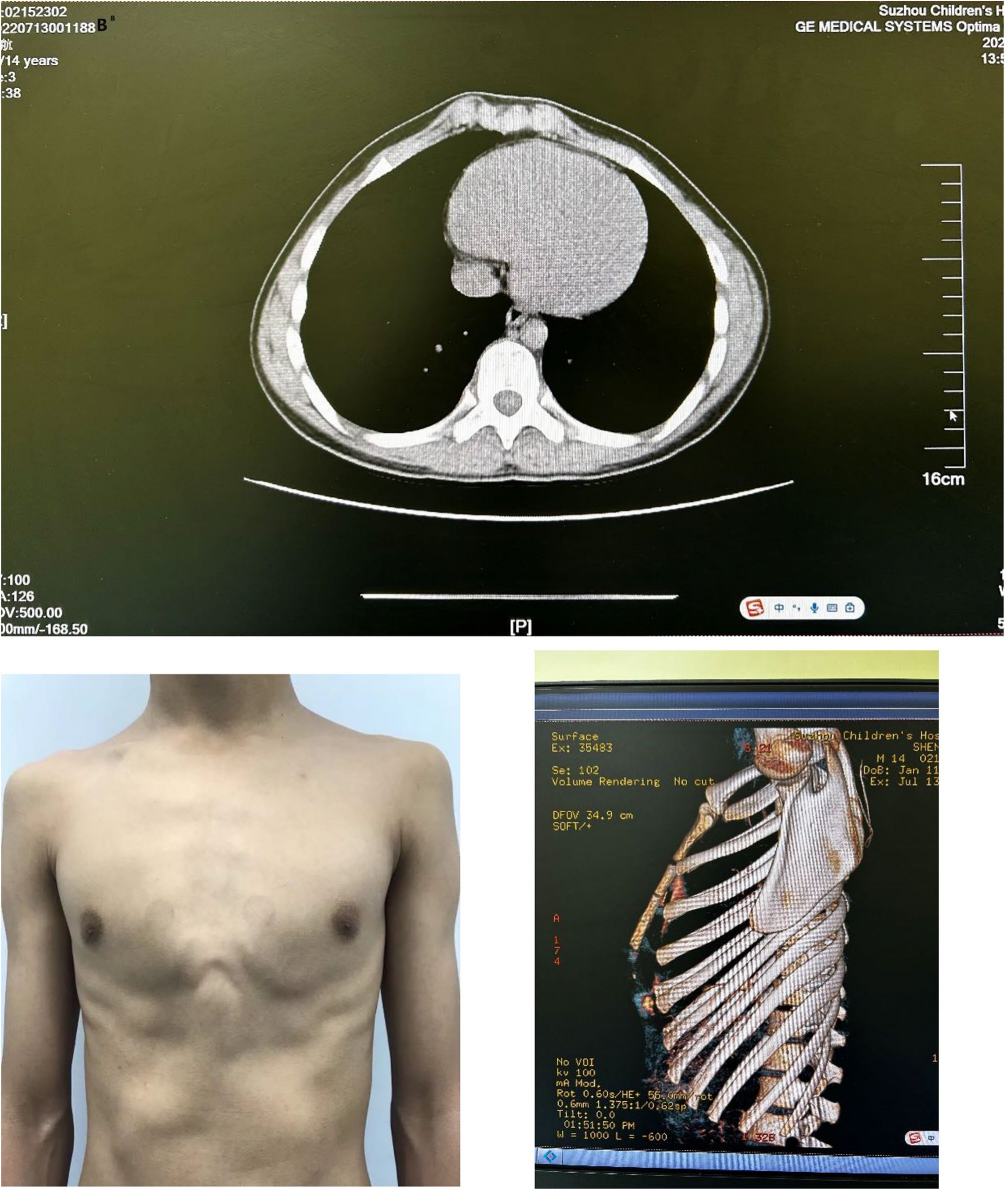
Chest appearance and CT scan of symmetrical PC

**Fig. 2:**
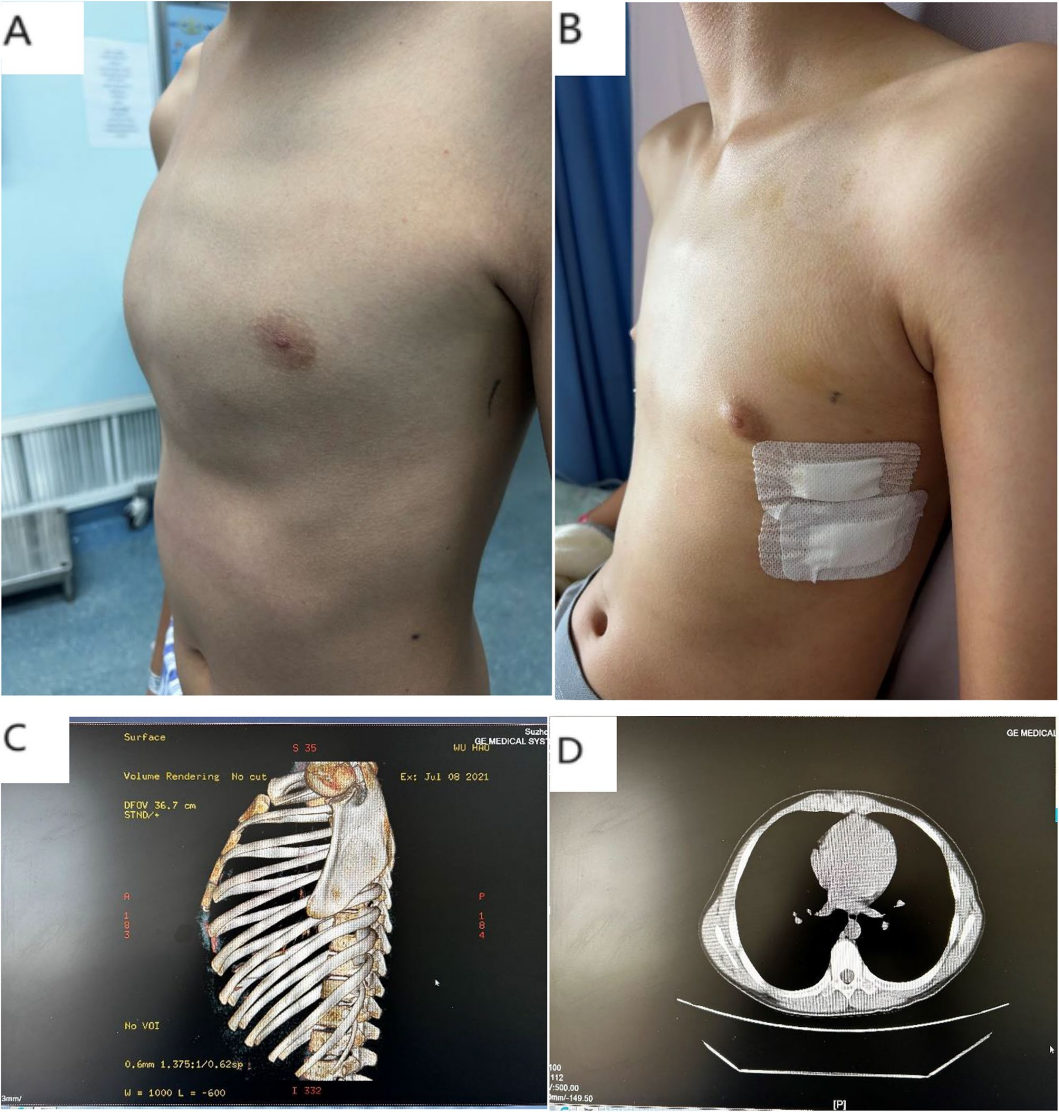
Chest appearance (**a**: preoperative, **b**: postoperative )and CT scan (**c**, **d**) of symmetrical PC

**Fig. 3:**
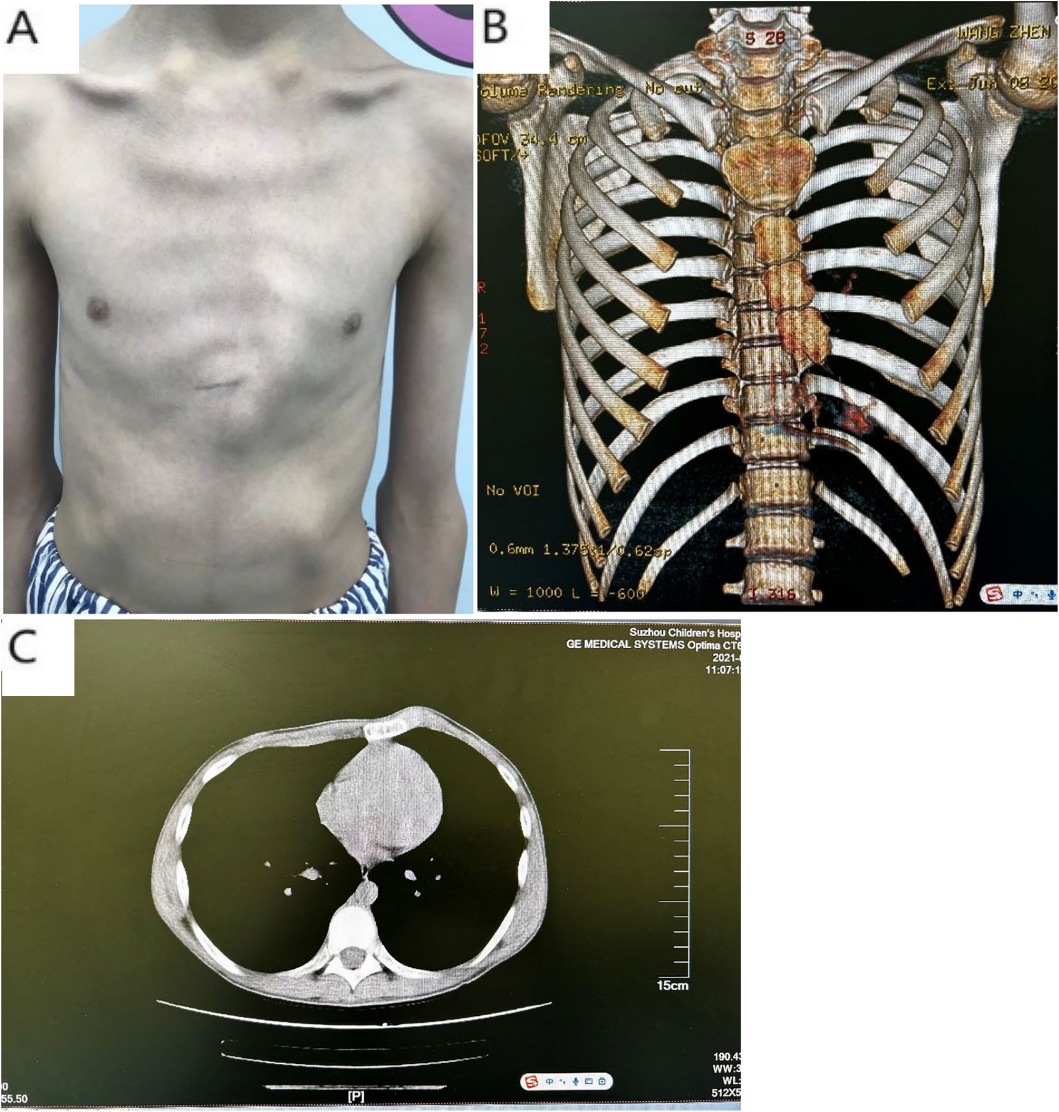
Chest appearance (**a**)and CT scan(**b, c**) of asymmetrical PC.B and C show the tilted and rotated sternum

Pectus carinatum (PC)mainly refers to the sternum and costal cartilage protruding forward strikingly and due to the overgrowth of costal cartilages and the adjacent ribs [1] and the metabolic disorders or sternocostal cartilage premature. 25% of PC patients having family history indicates probably genetic predisposition [2]. The PCis the second most common congenital anterior chest wall deformity and often found during the adolescent growth spurt. A number of studies have shown that PC accounts for 6%∼22% of chest malformations. It is estimated that the incidence of PC is 0.06% among all live born infants, about 1/1000 among adolescents, and about 4:1 between male and female. Whether or not symmetry, the deformity is divided into two types mainly : symmetrical PC (Figs. 1 and 2), asymmetrical PC (Fig. 3). and the sternum tilting to the right is more common in asymmetrical cases. The costal cartilage is prominent on one side, the contralateral side is sunken. This deformity may be best called PC and PE [3–5].

Salim Ramadan’s studies have shown that 29% of PC patients have thoracic symptoms at rest or when having physical activity without correlation with severity of deformity. Conceivable, the symptoms are related with exercise deconditioning for embarrassment to undress in public. 15% of PC patients presented chest pain, possibly is related to unusual muscular and nerve insertion [6]. The PC not only has a great impact on patients physically, but also can result psychological disorders to different extent [7].

## Patients and methods

### Patients

We retrospectively reviewed records of PC patients who underwent the surgical correction at our department between January 2020 and April 2023. This study recruited patients aged between 10 and 17 years old. The study was conducted in accordance with the Declaration of Helsinki. Written informed consent was obtained from their parents. Our study was approved by the ethics committee of Children’ s hospital of Soochow University(2,020,052).The bars we used are Nuss bar(Biomet Microfixation, Florida, U.S).

### Methods

The median age was 13 years (range10 to17 years). The examinations of all patients were performed Preoperatively. The laboratory tests, chest X-ray and Computed tomography (CT), spinal X-ray, electrocardiography (ECG), echocardiograph (ECHO)and cardiopulmonary function (CPF). We compress the protruding area manually to evaluate the chest wall flexibility preoperatively, so-called “fist test” [8]. Compression Depth (CD: difference value of the distance between the most protrusion point and the same point of the ninth vertebrae midpoint by lateral chest X-ray) is measured before and after operation.

### Indications

The age of all patients is over ten years (the female patients should be delayed to repair). The children or the parents are dissatisfied about the chest appearance. Primary complaints for surgery in PC patients are cosmetic nature and impact on patient’s self-image [2, 9, 10]. In most symmetrical PC cases, CT index (Haller index, HI) being smaller than 2.3 is indication for surgery. While in asymmetrical deformities or PC/PE group, HI is not the surgical indication, we rely on the outer shapes and CT 3-D technology mainly (Figs. 2 and 3).

### Surgical ways

The operation was performed under intravenous general anesthesia and tracheal intubation. The patients were in a supine position with bilateral upper extremity abduced. We marked the most protruding area and bilateral anterior axillary line incisions. The vital is to locate two adjacent ribs securing the bar by steel wires. We selected Nuss bars and shaped based on the wanted thoracic cage. Made transverse incisions which are from subcutaneous tissue to surface of ribs. Suture the absorbable line encircling the two adjacent ribs separately for a introducer to place steel wires. Make the subcutaneous tunnel by a long curved forceps on right side while by a polyvinyl chloride (PVC) tube with needle core on left side, which meet at the highest protruding area, then we remove the needle core and drag the tube to right side incision. The one end of the bar putted into the left side of the tube, drag the tube and the bar pass through the subcutaneous tunnel then flipped it. Pressed the highest point of the sternum for fixing two ends of bars to the ribs firmly with the preplaced steel wires. All the procedures done, the wanted chest appearance should be achieved. Nevertheless, patients in PC/PE group and the most asymmetrical will present the overcorrection lower plane secondary to the Abramson procedure. We decided to do the Nuss procedure to elevate it. Generally, we use thoracoscopic guidance to place the Nuss bar ensuring safety. Then we placement of drainage tube into right pleural cavity normally when we do the Nuss Procedure.

### Postoperative analgesic and care

**Fig. 4:**
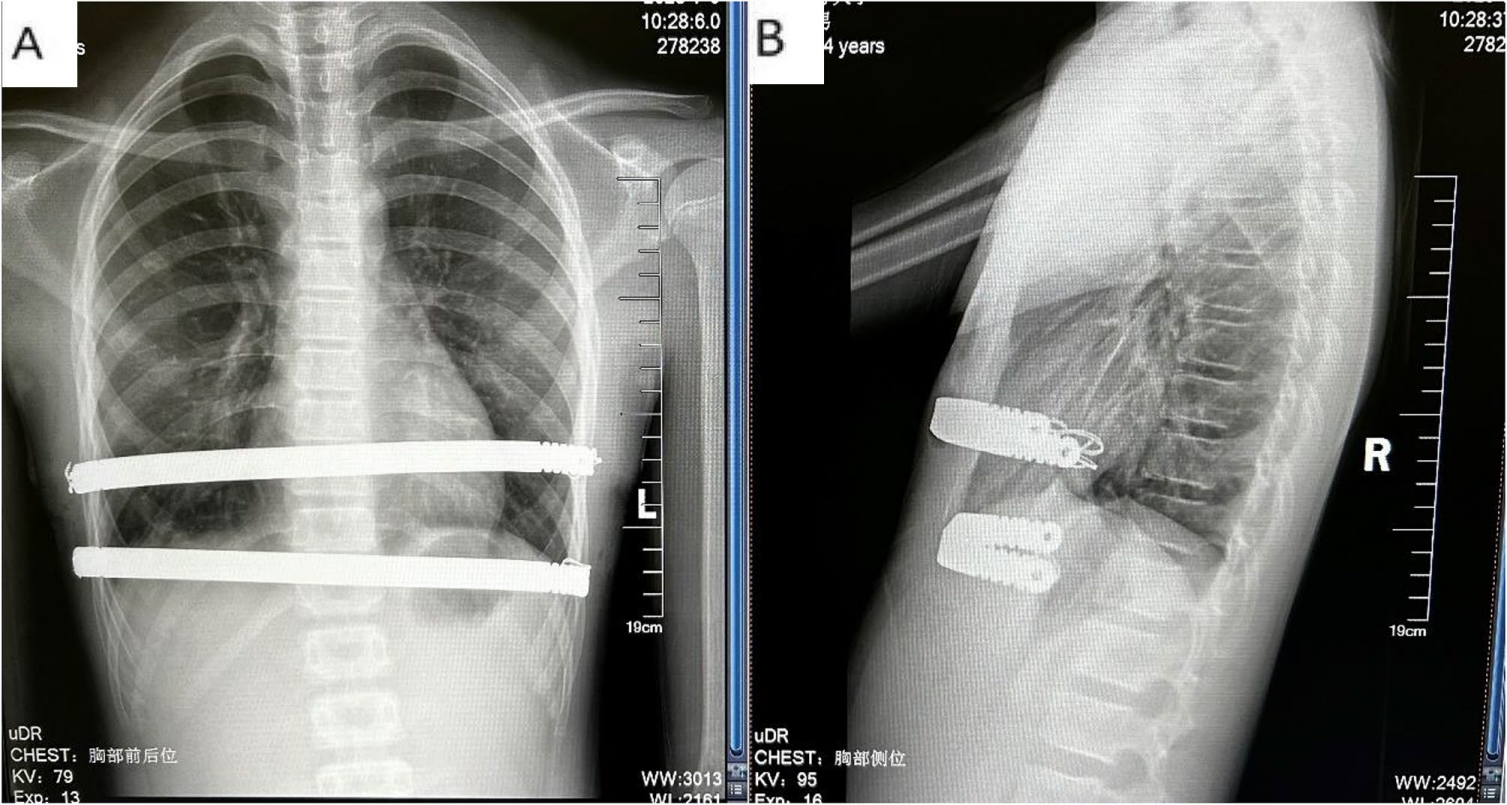
The chest X-ray of the postopertative in PC/PE group (**a**: posteroanterior, **b**: lateral)

**Fig. 5:**
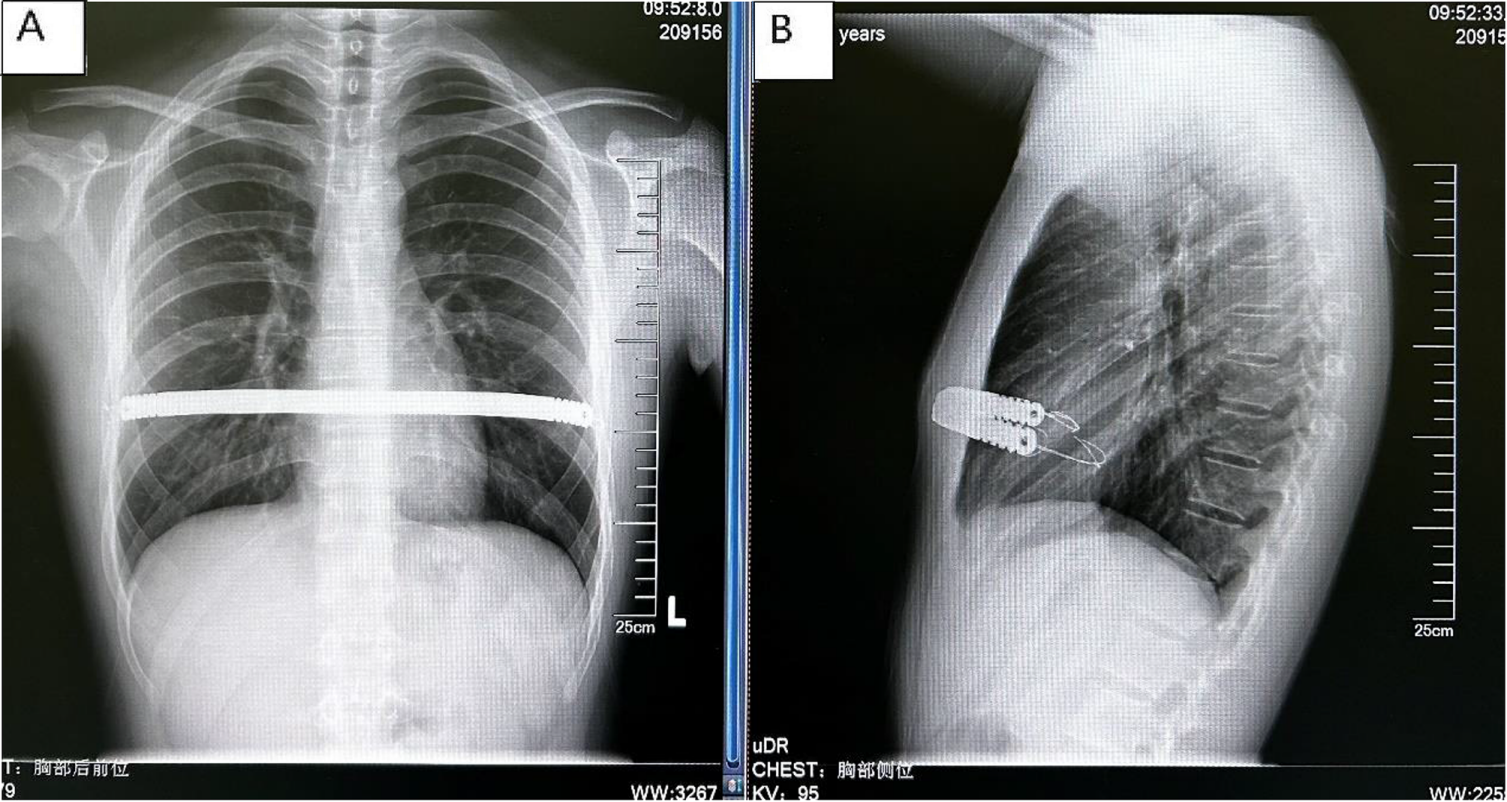
The chest X-ray of the postopertative in PC group (**a**: posteroanterior, **b**: lateral)

Ultrasoundguided serratus anterior plane block or ultrasoundguided thoracic paravertebral block(T5-7) were used before operation. Postoperatively, Pentazocine intravenous infusion and oral ibuprofen (Johnson & Johnson ) are for relieving pain to get sufficient mobilization. The first day after operation, physical therapies started. We persuade children patients to stand against to wall, walk and breath excise (deeply inspire) try the best. Patients were given a chest X-ray to find pneumothorax or hydrothorax. All patients have a one and a half years follow up. The examinations were performed to all the patients after operation: chest and spinal X-ray (Figs. 4 and 5). The follow up time points are the first month, the third month, the sixth month, one year postoperative and the last (one and a half years). The bar is removed after the end of follow up time.

### Evaluation of results after surgery

Satisfaction from questionnaires of their patents was divided into four grades:

Excellent: the outer shape looks normal, they were very satisfied with the surgery result.

Good: the outer shape was improved significantly, they were satisfied with the surgery result.

Fair: the outer shape was improved, they can accept the surgery result.

Poor: the outer shape was not improved, they cannot accept the surgery result.

### Statistical analysis

SPSS 17.0 statistical software were used for data analysis. Basic information including: sex, age, weight, height, type of operation and HI. We mainly evaluated the outcomes of two groups as follows: HI, symmetry, scoliosis, operation time, length of stay (LOS) and CD. Normally distributed continuous data were expressed as mean ± standard deviation. The comparisons among normally distributed continuous variables were conducted via t-test.

## Research results

### Demographics

From July 2020 to July 2023, 41 patients including 30 PC patients and 11 PC/PE patients were treated by Abramson procedure (PC)or Abramson procedure and Nuss procedure (PC/PE) in our center. We divided the cases into two groups: the PC group and the PC/PE group. There are 29 males and 1 female in PC group,11males in PC/PE group. The mean age was 162.13 ± 14.53 months in PC group and 162.09 ± 14.17 months in PC/PE group, with a median age of 156 months in PC group and 164 months in PC/PE group. The mean height was 167.67 ± 8.26 cm in PC group and 169.09 ± 7.69 cm in PC/PE group, with a median height of 168 cm in PC group and 169 cm in PC/PE group. The mean weight was 46.40 ± 6.91 kg in PC group and 45.27 ± 6.23 kg in PC/PE group, with a median weight of 45 kg in PC group and 46 kg in PC/PE group. The mean HI value was 2.02 ± 0.27 in PC group and 2.2 ± 0.32 in PC/PE group, with a median value of 1.9 in PC group and 2.2 in PC/PE group. There were 13 asymmetrical cases with the proportion to 43.3% in PC group. There were 7 asymmetrical cases with the proportion to 63.6% in PC/PE group. There were 7 cases having scoliosis with the proportion to 23.3% in PC group and 6 cases having scoliosis with with the proportion to 54.5% in PC/PE group. Two of them have scoliosis(Cobb angle ≥ 20°) need treat. One of them accepted brace treatment six months later postoperative and the angle is improved to 15°.The another(Cobb angle:19°) has no any treatment and lost follow. The mean operation Time was 77.90 ± 16.45 min in PC group and was 138.45 ± 43.44 min in PC/PE group, with the median time of 80 min in PC group and the median time of 130 min in PC/PE group. The mean LOS was 4.40 ± 0.72 days in PC group and was 5.18 ± 1.25 days in PC/PE group, with the median LOS was 4days in PC group and 5 days in PC/PE group. The mean CD was 32.87 ± 13.62 mm in PC group and was 18.27 ± 11.59 mm in PC/PE group, with the median depth was 32 mm in PC group and was 12 mm in PC/PE group. Postoperatively, there are some complications. Complications were stratified according to Clavien-Dindo classification (Table 1). Nine marginal pneumothorax patients being treated conservatively were found in five patients in PC group and in four patients in PC/PE group. One patient was found overcorrection when follow up one month later. The thorax cage collapsed transversely along with the bar. So we removed the bar and the PC was recurrent. There were 3 patients having steel wires breakage in PC group and 2 patients in PC/PE group. One of them had to reoperation postoperative three months later for dislocation of the bar secondary to the wires breakage. Up to now, 11 patients have removed the bars and have no recurrent case (Table 2: the end of article),10 patients (90.9%) achieved excellent or good results, 1 patients (9.1%) achieved fair results, and no patients had poor results and no metal allergy. The rest of 30 patients waiting for removal of the bar are excellent or good results in the follow time.

**Table 1:** Complications by patient

Complications(*n*, %)	Treatment	Clavien-Dindo grade
Pneumothorax (9,21.9)	no treatment	I
Overcorrection(1,3.3)	Surgery	IIIb
Steel wires breakage (5,12.2)	1 surgery 4 no treatment	IIIb I

**Table 2:** Comparison of surgical characteristics between PC group and PC/PE group

	PC group (*n* = 30)	PC/PE group (*n* = 11)	*P* value
Male/Female	29/1	11/0	
Age(mons)	156(162.13 ± 14.53)	164(162.09 ± 14.17)	0.993
Height(cm)	168(167.67 ± 8.26)	169(169.09 ± 7.69)	0.621
Weight(kg)	45(46.40 ± 6.91)	46(45.27 ± 6.23)	0.638
HI (CT)	1.9(2.02 ± 0.27)	2.19(2.2 ± 0.32)	0.078
Asymmetry (%)	13/30(43.3)	7/11(63.6)	0.249
Scoliosis (%)	7/30(23.3)	6/11(54.5)	0.057
operation Time(min)	80(77.90 ± 16.45)	130(138.45 ± 43.44)	0.001
LOS (days)	4(4.40 ± 0.72)	5(5.18 ± 1.25)	0.130
Compression Depth (mm)	32(32.87 ± 13.62)	12(18.27 ± 11.59)	0.003
Pneumothorax (%)	5(16.7)	4(36.4)	0.177
Overcorrection	1(3.3)	0	0.540
Steel wires breakage	3(10)	2(18.2)	0.487
Reoperation	1(3.3)	0	0.540
Removal of bar	8	3	0.969

## Discussion

The most patients seek treatment PC for aesthetic considerations with no significantly functional impairment. 26.3% of the patients had both cosmetic and health-related reasons to undergo surgery. Another 68.4% of PC patients sought surgical correction for solely cosmetic reason [11]. Dissatisfaction with own body image resulted in impaired self-esteem or may affect mental well-being as well, even to a life-threatening degree in more severe cases. Negative body attitudes and feelings have a stronger influence on suicidal ideation than depression, hopelessness, and past suicidal behavior [12]. Most patients in our center are also for the reason why they longed for increasing self-confidence and self esteem rather than the physical sick .

Over the past two decades, different techniques of corrective thoracic wall deformity have been used for repairing PC, such as the modified Ravitch, nonoperative bracing and minimally invasive methods [13–15]. Abramson procedure is the most popular MIR method of PC treatment recently [16, 17]. One study including a total of 396 patients after MIRPC by the Abramson method has showed satisfactory results in nearly all patients postoperative (99.5%, *n* = 183/184): high satisfaction rate of 91.0% (*n* = 190/209) after bar removal and low recurrence rates of 3.0% (*n* = 5/168) [15]. PC appears strikingly and was found mainly during the growth spurt time in the early teenage years or the puberty [11]. The male puberty begins at 12years old. In our study, the median age of two group patients correction for the deformity is 13years(156mons) and 13.7years(164mons), the results coincide with the fact. Their parents found that chest outer appearance changed strikingly when rapid growth at the beginning of puberty. Fortunately, the time of puberty(12years-18years) is the best age to repair PC by the Abramson method for the relatively high chest wall flexibility and decreased force to overcome during MIR [8, 18]. In our results, the 41 patients have a good results without severe complications except one overcorrection. Steel wires breakage presented in five patients, mainly for using the smaller (5#) steel wires because we underestimated the strength of the growth spurt. So, we fix the bars by bigger (6#,7#) steel wires after then. A tension-free subcutaneous tunnel and more favorable position of the steel wires are beneficial to avoid the accident. As to the pneumothorax, above all, the skillful operative techniques are critical. The reason is the pleural cavity and the intercostal muscles were broken when we placed steel wires ; and then the shortest distance between steel wires and the end of bar will attenuate the damage. With the shorter follow time and less patients, we have not experienced recurrence cases until now. Likelihood of recurrence is sure in future when more patients are treated. Some patients may be poor results because the elder age resulting in more stiff thoracic cage.

The median HI is 1.9 in PC group while 2.2 in PC/PE group and the former is more significant to repair take for granted. Meanwhile, the median compression depth is 32 mm in PC group while 12 in PC/PE group having statistical difference between in them(*P* < 0.01). In theory, the smaller value of HI, the deeper of the compression should be, the linear trend show the fact, but when the correlational analysis is conducted and found no correlation (*r*=-0.2071, *r*= -0.4122). when the patients are asymmetrical or complicated cases, the HI value cannot evaluate the surgical indication. The Abramson procedure is not enough to repair some asymmetrical and complicated PC patients. The reasons why that for the asymmetric or the PC/PE, the compression improve the local protrusion but result overpressure of the contralateral side, the more asymmetric, the overpressure more striking. The proximal sternum and the ribs is very strong and not prone to be compression, the distal part of the sternum easier descending excessively, dragging the low ribcage with it. So the Nuss procedure can prevent the overcorrection.

So the surgeons make decision on the basis of patient’s outer appearance comprehensively. In PC/PE group, the asymmetrical deformity is 63.6% (7/11) higher than the 43.3% (13/30) of the PC group though not statistical difference(*P* > 0.05). PE. Hyung Joo Park [17] and colleagues completed 58 cases PC by the similar procedures (they called Sandwich procedures). They concluded that the procedure seems to be effective in repairing asymmetric PC and complex deformities. As a result, asymmetry is one of the reason why surgeon use two procedures to repair the deformity.

The successful keys of this operation are the following aspects: (1) The sternal or costa-cartilage highest point is the stress power center of the plate. Therefore, plate fixations should be symmetrical as far as possible, so that the force on the plate is balanced and not prone to displace. (2) A loose and tension-free subcutaneous tunnel should be built to avoid unbalance power of the skin and muscles. (3) The position of the steel wires used to fix the bar should be placed at the nearest neighbor ribs so as to minimize the displacement and instability of the plate caused by the retraction. (4) Decision-making to the number of bar : with sole lower sternum or costa-cartilage protrusion, The modified Abramson procedure is enough; with obvious asymmetry and presence of a carinatum and excavatum component, the Nuss procedure is also needed. (5) Good pain control and frequent mobilization (get out of bed early (first day after operation) are essential postoperatively.

### Limitations

There are some limitations. In this paper, where need to be improved are the limited duration of follow-up and patient number. This study is a retrospective observational study. Though the outcomes of the procedure seem promising. In the future, we will summarize more cases and closely scrutinized for a longer-term outcomes.We should test pulmonary function postoperatively, and give analysis of the different value before and after operation to find the compression whether or not compromise pulmonary function.

## Conclusions

In this study, the surgical outcomes of MIR(Abramson procedure or Abramson couple with Nuss procedure) are sure without severe complications. CD value may be an indicator evaluating surgical results of the symmetrical PC. The thoracic deformity of PC is various, the surgical procedure selection should be individual. In PC/PE group and the asymmetrical, patients treated by Abramson procedure and Nuss procedure, combination of two procedures makes up for the deficiency of the sole Abramson procedure.

## Data Availability

No datasets were generated or analysed during the current study.

## Abbreviations

PC: Pectus carinatum
PE: Pectus excavatum
CT: Computed tomography
HI: Haller index
MIR: Minimally invasive repair
PVC: Polyvinyl chloride
CD: Compression depth
